# Thoracic splenosis presenting as pulmonary space-occupying lesion

**DOI:** 10.1186/s12893-018-0461-9

**Published:** 2018-12-20

**Authors:** Yumeng Niu, Wenzhou Liu, Lei Xian, Tao Liu, Chusheng Huang, Shengzhuang Yang

**Affiliations:** Department of Cardiothoracic Surgery, The Second Affiliated Hospital of Guangxi Medical University, No.166 Da Xuedong Road, Nanning, 530007 Guangxi China

**Keywords:** Spleen, Thoracic surgery, Lung, Thoracic splenosis

## Abstract

**Background:**

Spleen leaves its normal anatomical position and appears in other locations, which is called ectopic spleen. It is most commonly found in the abdomen or pelvis with seeding of the peritoneum, omentum or mesentery. A few of cases of thoracic splenosis associated with traumatic diaphragmatic rupture have been reported.

**Case presentation:**

We make a report on a case of intrapulmonary thoracic splenosis. A 44-year-old male patient underwent splenectomy due to a high fall accident injury in 2008. After ten years, thoracic splenosis were found in the lungs and chest wall. Clinical diagnosis was unidentified masses, benign tumor of lungs and chest wall. The radiological imaging was suggestive of the thoracic splenosis, After surgery, the diagnosis of thoracic splenosis was confirmed by pathological diagnosis.

**Conclusions:**

Thoracic splenosis may occur after the injury to spleen and surgical treatment may not be the preferred method for asymptomatic or less symptomatic thoracic splenosis.

## Background

Splenosis is the autotransplantation of splenic tissue following spleen rupture or splenectomy. Despite the relatively high incidence of splenic trauma, splenosis is still a rare condition. Ectopic spleen is a condition that spleen leaves its normal anatomic position and implants in other location such as a serosal surface, deriving a local blood supply and developing into nodules of differentiated splenic tissue [1]. To our best knowledge, we only have identified a few of well-documented cases in the literature [2–4]. Although heterotopic spleen is widely reported in literature, thoracic splenosis that presents as a pulmonary lesion has not been well-known.

## Case presentation

**Fig. 1:**
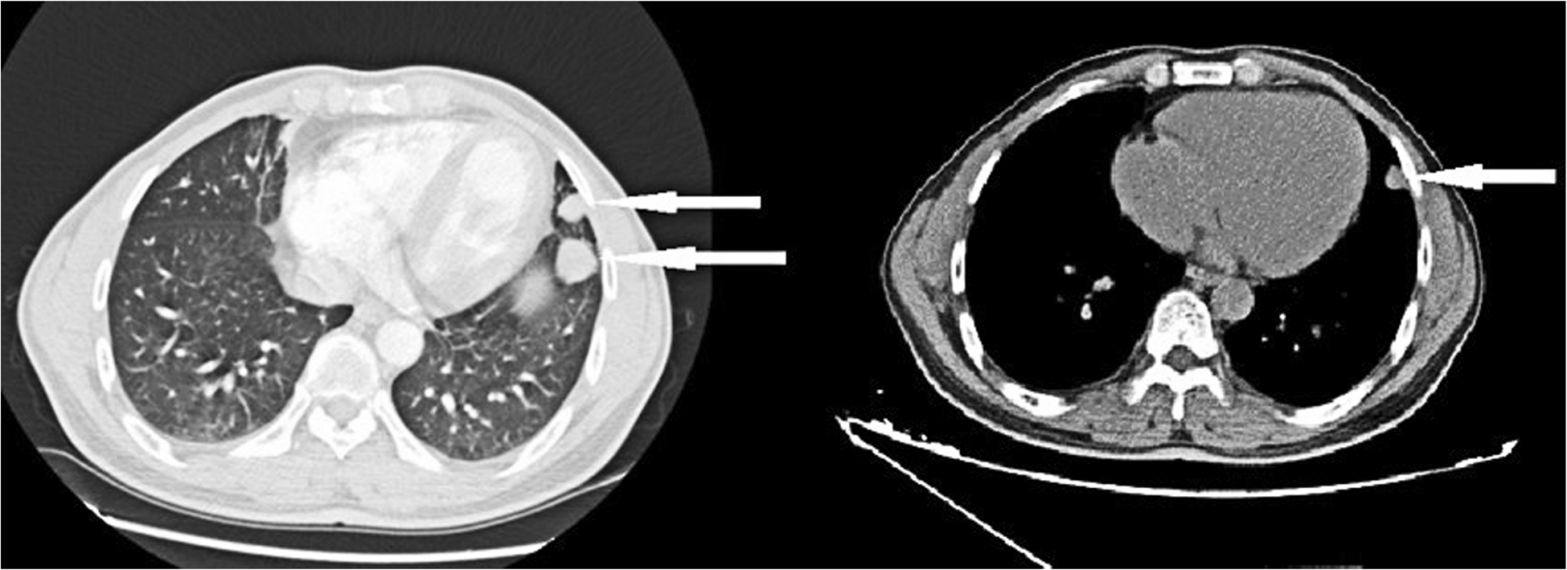
computed tomography scan (white arrow) of the chest showing the occupancy of mediastinum and the lungs

44-year-old man had a history of trauma and splenectomy dating back to 2008 due to a high fall accident injury. The patient came to our hospital for routine follow-up health check for trauma. During the interview with the doctor, the patient had complaints of fatigue, flu-like symptoms and occasionally had the sense of thoracalgia for about a week. During the physical examination, a well healed laparotomy scar measuring about 10 cm in the upper left abdomen was identified. No spleen was palpable when palpation of left costal region was conducted by the doctor. No other significant findings were identified. All laboratory findings were within normal range. Hematology and biochemistry tests were within normal range. All other laboratory findings were within normal range too. Pulmonary function tests and cardiovascular examination showed the normal state. Plain chest computed tomography (CT) with an attenuation value 52HU was performed. There were multiple nodules under the left upper lobe tongue segment, lower lobe basal segment subpleural and right diaphragmatic, partial fusion showed wavy changes with uniform density. There were clear boundary and enhanced scanning lesions, measuring up to 18 mm, suggesting suspicious for primary lung cancer. There were old fractures of the left side 9th and 10th ribs, and no right sided pulmonary lesions or mediastinal lymphadenopathy were seen on CT. Contrast-enhanced CT scans showed obvious enhancement in soft tissue masses (Fig. 1).

**Fig. 2:**
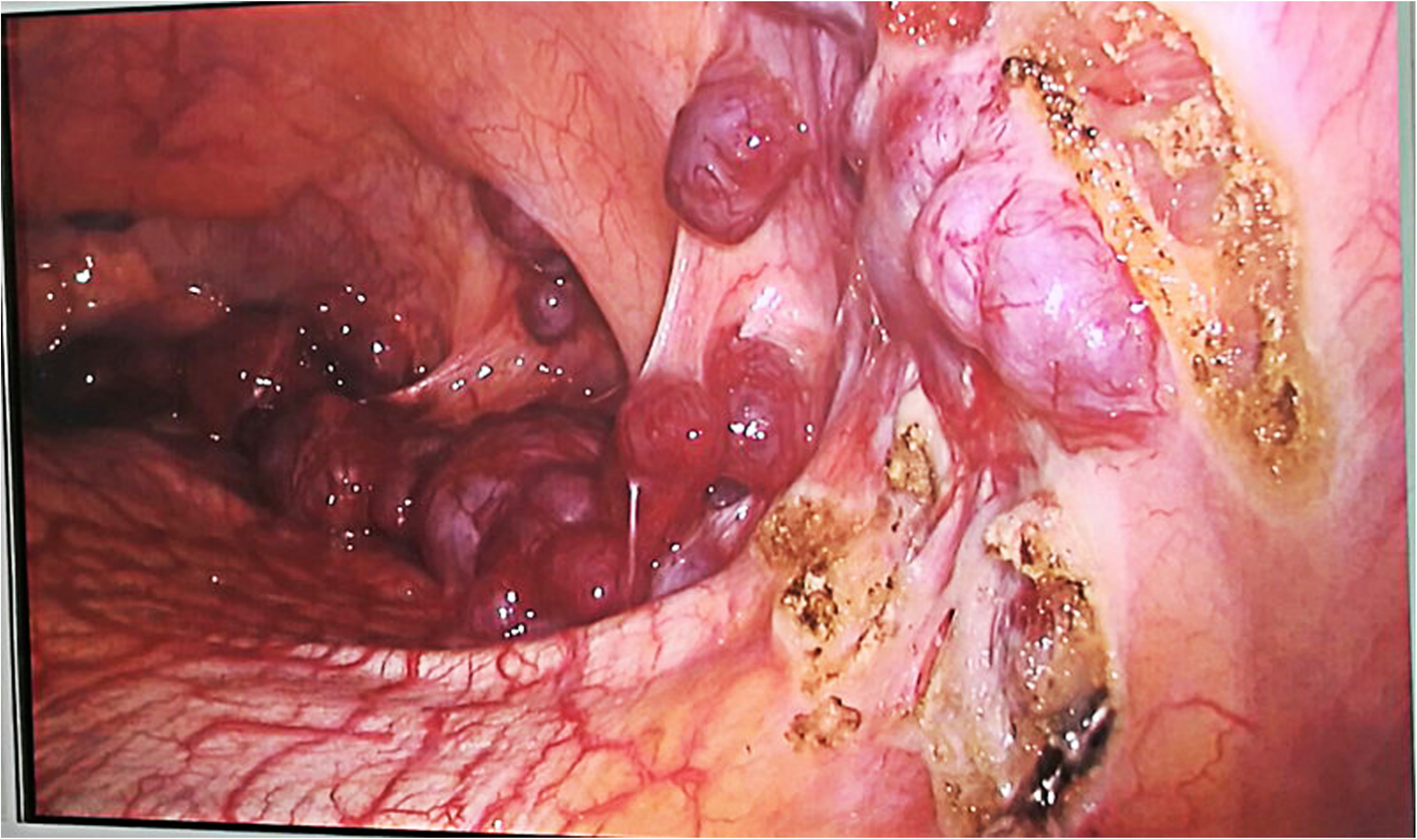
large number of botryoid masses on diaphragm and pleural in left thoracic cavity

Based on CT imaging, the surgery of left thoracic exploration and lumpectomy of the chest wall was planned to relief symptom, remove masses. After discussed with patient. Via video-assisted thoracic surgery (VATS) was performed to remove the masses. During the operation, we found mass on the surface of the upper lobe of the left lung, and a large number of nodules that scattered on the surface of left lung surface, diaphragm and mediastinal pleura. The largest nodule was tough in texture, regular in shape, and was measured about 1.82.0 cm on pleura. (Fig. 2) We completely removed this biggest mass and other masses on left pleura through thoracotomy. After careful hemostasis, a chest tube was placed in the seventh intercostal space at the mid-axillary line and attached to an underwater seal. The left lung was re-inflated under direct vision. On the third day after surgery, the chest drain was removed after minimal drainage.

**Fig. 3:**
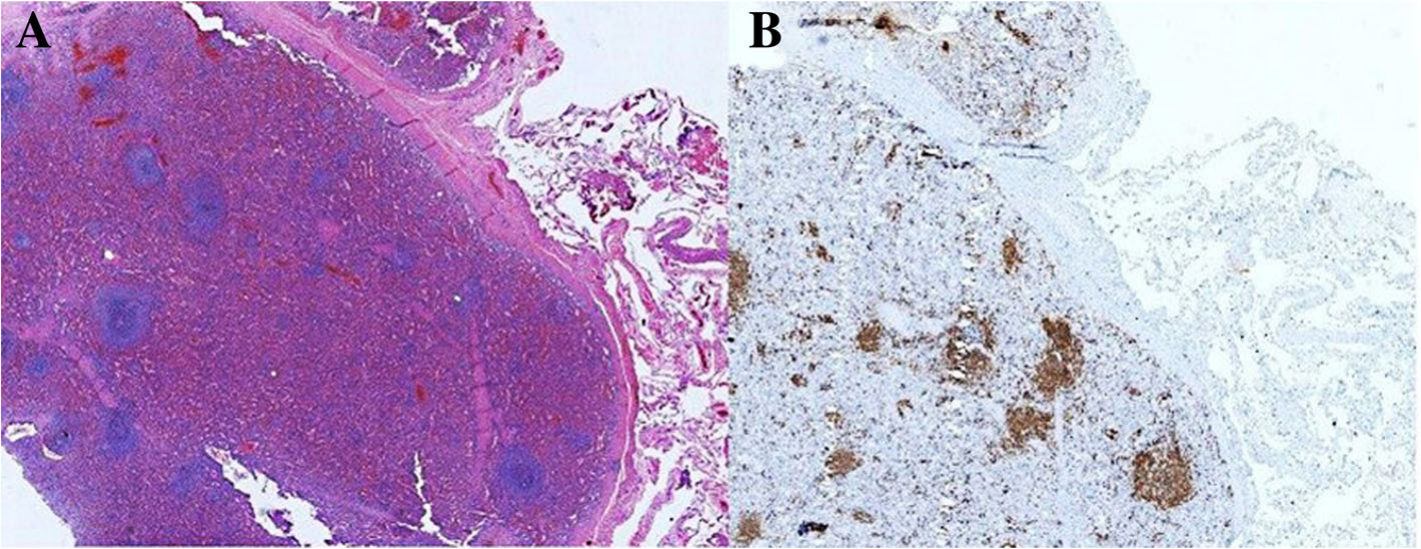
Pathology of left thoracic mass: (**a**) Hematoxylin and eosin staining slide at *400 magnification. The cells of thoracic mass showed the structure of spleen that was adjacent to the lung tissue. (**b**) immumohistochemical stain at *400 magnification. Cells of masses were CK(−)、CD3(T cell+)、CD20(B cell+)、TTF-1(−)、Syn(−)、Ki-67(+,5%)、CD34(sinusoid+)、CD8(sinusoid+)、CD1a(−)、S-100(−)、CD68(sinusoid+)

The patient had an excellent recovery after surgery and was discharged properly. Final pathological findings: (Fig. 3) on H&E slide, the mass was consisted of red pulp and white pulp of spleen with splenic corpuscle and spleen trabecular structure, which showed viable splenic tissue. Immunohistochemically, cells of masses were CK(−)、CD3(T cell+)、CD20(B cell+)、TTF-1(−)、Syn(−)、Ki-67(+,5%)、CD34(sinusoid+)、CD8(sinusoid+)、CD1a(−)、S-100(−)、CD68(sinusoid+) (Fig. 3). The final pathological diagnosis was splenosis without malignancy.

## Discussion and conclusion

Till now, there were only a few numbers of publications reporting perforation of thoracic splenosis that presents as pulmonary lesion. Trauma is currently considered to be the main cause of splenic implantation. Previous report introduced multiple ectopic splenomegaly in the abdominal cavity and pelvic cavity after splenectomy due to trauma [5]. Thoracic splenosis is an uncommon clinical entity and only accounts for less than 0.25% of splenectomies. Because conservative treatment for an asymptomatic splenosis is associated with a complication rate of 65%, [6] the most treatment preferred for Thoracic splenosis is operation, including splenopexy or splenectomy, in uncomplicated and complicated cases. The vast majority of patients with heterotopic splenic tissues were characterized mainly as splenosis in abdomen or digestive system after splenectomy. In this case, although the previous operation notes at the time of the splenectomy did not mention any “escaped” spleen, the patient experienced splenic trauma with likely perforation of the left hemi-diaphragm and seeding of splenic tissue into the left thoracic cavity, which is most likely to be the source of thoracic splenosis.

Splenic implantation often occurs in the abdominal cavity, and often be misdiagnosed as malignant tumor of digestive system, such as liver cancer, gastric leiomyoma at the bottom, or mesothelioma. Ectopic spleen to the chest can be misdiagnosed as lung cancer [7]. Thoracic splenosis usually presents as an incidental finding several decades after splenic trauma and rarely causes symptoms. In this case, the patient came to hospital for follow up check-up, further examination revealed masses in chest cavity, therefore, the thoracic splenosis in this case may be considered as incidental findings. Further surgery that was performed to remove splenosis may relief patient’s symptoms he already had for weeks.

Ultrasound, CT, and MRI etc. The diagnosis of thoracic splenosis cannot be made clearly through ultrasound, CT, or MRI, etc [8]. Digital subtraction angiography (DSA) can show heterotopic spleen with an independent blood supply. Three-dimensional angiographic can clearly display the celiac axis or abdominal aorta main blood vessel, which provide great helps diagnosing ectopic splenic vascular remodeling. However, the structure of spleen-implanted reticular spleen-trabeculae was significantly reduced. There was no hilus of spleen and the blood supply came from the small vessels in the thoracic splenosis. Therefore, the diagnosis of thoracic splenosis may not be made through DSA. Splenosis on pleural may be misdiagnosed for an intrathoracic malignancy, which usually triggered prompted invasive investigation with needle or VATS biopsy. Previous reports suggested that when thoracic splenosis was suspected, the diagnosis may also be made on 99 m-Technetium heat-damaged erythrocyte scan, which has a high sensitivity and specificity for splenic tissue [9]. A previous history of splenectomy would also be helpful for diagnosis. The incidence of heterotopic spleen is high in abdominal cavity and pelvic cavity, and splenic torsion could be easy to occur in wandering spleen [10]. Most of the patients with thoracic splenosis had abdominal discomfort, but most of them had no obvious clinical symptoms. Clinical symptoms appear only when there was a twist or a pathological spleen. Surgical resection through laparoscopy is the main treatment for this type of heterotopic spleen [11, 12]. However, surgical resection is not the first choice for multiple ectopic spleens. Small, asymptomatic thoracic splenosis can be monitored, but large ones that caused unendurable symptoms should be removed by surgery. Although the histology of thoracic splenosis resembles normal splenic tissue, it is still associated with reduced immune function as the residual volume and function is insufficient to confer protection against overwhelming post-splenectomy infection. Therefore, in principle, the implantation of asymptomatic thoracic splenosis may not necessarily be removed by surgery but the immunization and early prophylactic penicillin remains needed. Finally, a clear preoperative diagnosis is required to avoid unnecessary surgery [13].

## Abbreviations

CT: Computed tomography
MRI: Magnetic resonance imaging
DSA: Digital subtraction angiography
